# Renal biomarkers are less useful at predicting acute kidney injury in patients with sepsis than those without

**DOI:** 10.1186/cc10380

**Published:** 2011-10-27

**Authors:** N Glassford, A Schneider, G Eastwood, L Peck, H Young, M Westerman, V Ostland, R Bellomo

**Affiliations:** 1 Department of Intensive Care, Austin Hospital, Heidelberg, VIC, Australia; 2 Intrinsic LifeSciences LLC, La Jolla, CA, USA

## Introduction

Multiple biomarkers have been proposed for identifying patients at risk of developing the syndrome of acute kidney injury (AKI) [1]. These biomarkers include urine and serum NGAL, and urinary hepcidin. The pathophysiology of AKI in sepsis appears to be primarily mediated by immunological, toxic and inflammatory factors as opposed to renal ischaemia [2]. Different aetiologies of AKI are likely to lead to differential release of serum and urinary biomarkers. We sought to determine if the predictive ability of several renal biomarkers for predicting AKI varied in the presence of sepsis in the context of routine ICU practice.

## Methods

We measured serum and urinary NGAL and urinary hepcidin in patients admitted to the ICU of a tertiary referral hospital with SIRS and either oliguria or a 25 μmol/l serum creatinine increase within 48 hours of admission. We used point-of-care creatinine measurements to identify the maximum RIFLE category of AKI within the first 5 days of enrolment. We corrected both urinary biomarkers for urinary creatinine. We calculated the reciprocal of hepcidin measurement and noted if serum NGAL was greater than the upper limit of normal (149 ng/ml). We derived the area under the curve (AUC) for the receiver operating characteristic curve (ROC) for all biomarkers.

## Results

Between 31 August 2010 and 17 November 2010, we enrolled 92 patients; 17 of these patients had APACHE II diagnoses of sepsis. In patients with a diagnosis of sepsis, the predictive ability of all of the biomarkers measured was worse than in those without (Table 1).

**Table 1:** AUC ROC for the prediction of AKI

	ROC AUC							
	
	RIFLE R, I or F				RIFLE I or F			
		
	Septic		Nonseptic		Septic		Nonseptic	
				
Test result variable	Area	SE	Area	SE	Area	SE	Area	SE
Urinary NGAL	0.367	0.136	0.561	0.068	0.367	0.136	0.633	0.090
Urinary NGAL corrected for urinary creatinine	0.417	0.136	0.578	0.066	0.417	0.136	0.670	0.082
Serum NGAL	0.375	0.162	0.639	0.065	0.375	0.162	0.685	0.087
Serum NGAL positivity	0.492	0.158	0.611	0.066	0.492	0.158	0.674	0.082
Urine:serum NGAL ratio	0.483	0.140	0.498	0.068	0.483	0.140	0.543	0.081
1/urinary hepcidin	0.508	0.153	0.624	0.066	0.508	0.153	0.611	0.080
1/urinary hepcidin corrected for urinary creatinine	0.483	0.156	0.598	0.067	0.483	0.156	0.578	0.083

SE, standard error.

## Conclusion

Although the sample size is limited, there is a marked difference in the predictive ability of the measured biomarkers to predict AKI between septic and nonseptic patients. All patients admitted met the criteria for a diagnosis of SIRS, suggesting that inflammation and sepsis contribute to the development of AKI via different pathways. The ability of these biomarkers to predict AKI in patients with a diagnosis of sepsis in our cohort is limited. Further investigation is needed into whether the combination of specific biomarker patterns and clinical features can better identify patients at risk, particularly in the setting of sepsis. In addition, further work examining the relationship between the various biomarkers and the aetiology and natural history of AKI is required.
